# Natural Products from Antarctic Colonial Ascidians of the Genera *Aplidium* and *Synoicum*: Variability and Defensive Role

**DOI:** 10.3390/md10081741

**Published:** 2012-08-20

**Authors:** Laura Núñez-Pons, Marianna Carbone, Jennifer Vázquez, Jaime Rodríguez, Rosa María Nieto, María Mercedes Varela, Margherita Gavagnin, Conxita Avila

**Affiliations:** 1 Department of Animal Biology (Invertebrates), Faculty of Biology, University of Barcelona, Av. Diagonal 643, Barcelona 08028, Catalunya, Spain; Email: jenvazk@gmail.com (J.V.); conxita.avila@ub.edu (C.A.); 2 Institute of Biomolecular Chemistry, CNR, Via Campi Flegrei 34, Pozzuoli I-80078, Napoli, Italy; Email: mcarbone@icb.cnr.it (M.C.); mgavagnin@icb.cnr.it (M.G.); 3 Department of Fundamental Chemistry, Faculty of Science, Campus Zapateira, University of La Coruña, A Coruña 15071, Spain; Email: jaime.rodriguez@udc.es (J.R.); rnieto@udc.es (R.M.N.); 4 Department of Marine Science and Applied Biology, University of Alicante, Carretera San Vicente del Raspeig s/n, Alicante 03690, Spain; Email: varelamercedes@gmail.com

**Keywords:** Antarctic colonial tunicates, deterrent activity, sea star *Odontaster validus*, amphipod *Cheirimedon femoratus*, antibacterial activity

## Abstract

Ascidians have developed multiple defensive strategies mostly related to physical, nutritional or chemical properties of the tunic. One of such is chemical defense based on secondary metabolites. We analyzed a series of colonial Antarctic ascidians from deep-water collections belonging to the genera *Aplidium* and *Synoicum* to evaluate the incidence of organic deterrents and their variability. The ether fractions from 15 samples including specimens of the species *A. falklandicum*, *A. fuegiense*, *A. meridianum*, *A. millari* and *S. adareanum* were subjected to feeding assays towards two relevant sympatric predators: the starfish *Odontaster validus*, and the amphipod *Cheirimedon femoratus*. All samples revealed repellency. Nonetheless, some colonies concentrated defensive chemicals in internal body-regions rather than in the tunic. Four ascidian-derived meroterpenoids, rossinones B and the three derivatives 2,3-epoxy-rossinone B, 3-epi-rossinone B, 5,6-epoxy-rossinone B, and the indole alkaloids meridianins A–G, along with other minoritary meridianin compounds were isolated from several samples. Some purified metabolites were tested in feeding assays exhibiting potent unpalatabilities, thus revealing their role in predation avoidance. Ascidian extracts and purified compound-fractions were further assessed in antibacterial tests against a marine Antarctic bacterium. Only the meridianins showed inhibition activity, demonstrating a multifunctional defensive role. According to their occurrence in nature and within our colonial specimens, the possible origin of both types of metabolites is discussed.

## 1. Introduction

Ascidians are exclusively marine animals, occurring in all oceans, with >2800 described species [1]. They may be solitarian, or constitute social groups of individuals connected by the base, or be compound (colonial), with many clonal zooids embedded in a gelatinous matrix sharing the external tunic [2]. This outer integumentary tissue, harbors diverse cell types, including symbionts in some cases, and it is multifunctional, exhibiting variable consistency, from gelatinous to leathery [3]. Ascidians are sessile ciliary-mucus filter feeders, of which natural dispersal is almost exclusive of gamete and larval stages. This is not usually more than a few meters, especially in colonial species which produce fewer but larger eggs rich in vitelum, then forming lecitotrophic larvae that are brooded until released as tadpoles [1,2].

A great variety of predators feed on ascidians and these have evolved many mechanisms to prevent predation, mostly related to physical or chemical properties of the tunic [1,4]. Tough tunics occur in some colonial ascidians, but they are mainly found in solitarian ascidians [5]. Besides, calcium carbonate spicules embedded within the tunics of certain species may serve to avoid consumption [6,7,8]. Occasionally, palatability is more related to the nutritional value [4]. However, defensive chemistry is likely the first line of protection adopted by most ascidians. This may include the accumulation of heavy metals like vanadium, or sulfuric and (or) hydrochloric acid in tunic bladder cells [9,10,11,12]. However, the production of deterrent natural products is a common strategy too [8,12,13]. In certain species, these compounds are transferred from adults to larvae and eggs to confer protection, especially in compound ascidians where the investment in reproduction is particularly valuable [11,14,15]. Redundancy of protection through several defensive mechanisms can operate either against diverse enemies, or also at different life stages [4,8,11,12,16]. Indeed, clonal organisms, consisting of clumps of genetically identical, but independent individuals, more typically develop inducible distasteful chemicals, rather than solitary (aclonal) ones that are less likely to recover from a significant loss of tissue [17]. Furthermore, colonial ascidians tend to maintain a clean, unfouled surface, an indication of antifouling properties. Most of these mechanisms block initial bacteriofilms, avoiding further biofouling, epibiosis and infections [18]. Instead, a number of solitary species become heavily fouled and cryptic, which is a proposed tactic of hiding from possible enemies [1,9,19].

In 1974, Fenical isolated the first ascidian bioactive metabolite, geranyl hydroquinone from *Aplidium* sp. Since then, ascidians have yielded numerous compounds with remarkable bioactivities, including the first marine natural product to enter human clinical trials, didemnin B (reviewed in [20]). Ascidians mostly possess nitrogen-bearing metabolites, particularly aromatic heterocycles, like peptides, alkaloids, and amino acid derived products, but also non-nitrogenous compounds in lesser amounts, such as lactones, terpenoids or quinones [20,21]. Although the ecological function of most of these metabolites remains undetermined, it is known that at least some of them are used as predator deterrents [8,13,15,22,23] as well as antifoulants [24]. A number of bioactive natural products have been obtained from Antarctic ascidians coming from shallow as well as deep seafloors, such as palmerolide A, a group of ecdysteroids, meridianins, aplicyanins and rossinones [25,26,27,28,29]. It is often unclear if the animals are the true producers of the molecules [30,31,32] or if associated microbes may play a role in the secondary metabolism [33] (and reviewed in [34,35]). Indeed, microsymbiotic origin of ascidian metabolites has received much less attention [36] with respect to compounds from sponges (reviewed in [37]).

While the vast majority of ascidian metabolites have been isolated from whole-body extractions, several compounds were obtained from specific tissues, physiological fluids or cells [20,31,38,39,40]. If these products resulted in them possessing ecological defensive functions, then this particular location should be contrasted with the Optimal Defense Theory (ODT). The ODT predicts effective allocation of defensive compounds in most valuable/exposed body-regions of liable prey organisms, attending to the metabolic costs that entail secondary metabolite production [41]. Localization of defenses to specific regions has been observed in some sponges [42], and gorgonians [43], among other invertebrates. Ascidians possess a complex, organized body-plan and circulatory system, which may allow them to encapsulate bioactive compounds to fulfil ecological roles avoiding autotoxicity [44].

In Antarctic benthic ecosystems, invertebrate predators, mainly asteroids but also dense populations of amphipods, have replaced fish as principal predators [45,46,47]. Sea stars feed by extruding their cardiac stomachs over their prey, and initiating digestion from the outer layers [48], while amphipods bestow superficial bites. Hence, in most Antarctic organisms chemical defenses should likely be stored externally to benefit survival.

Ascidiacea is one of the principal taxa structuring Antarctic-shelf filter-feeding communities [49]. The ascidiofauna here is very homogeneous and endemic, with many species showing circumpolar as well as eurybathic distributions [50]. Within the Family Polyclinidae, one of the most prolific genera is *Aplidium*, with 40 species described from the Southern Ocean. *Synoicum* instead is represented by eight Antarctic and subantarctic species. *Synoicum adareanum* produces pedunculated colonies of variable colorations, whereas those of *Aplidium* are usually globular, with *A. falklandicum* being characteristically bright yellow, *A. fuegiense* pink-orange, *A. meridianum* gray, green, or brownish but with bright yellowish reflexes, and *A. millari* being mostly pink [51].

In this study, we aimed to evaluate the defensive potential based on the lipophilic secondary metabolism of several deep-water Antarctic ascidian species of the genera *Aplidium* and *Synoicum* to fight against sympatric predation and bacterial fouling. For this purpose we conducted feeding assays with the ether fractions of selected ascidian samples, using the asteroid *Odontaster validus* and the amphipod *Cheirimedon femoratus* as putative consumers, while considering the presumptions of the ODT in terms of intra-colonial defense allocation. Moreover, the antibiotic activity towards an Antarctic marine bacterium was also assessed. Finally, chemical analysis carried out on some of the samples led to the purification of several characteristic compounds, which were similarly tested for their defensive ecological activities.

## 2. Methods and Materials

### 2.1. Collection of Samples

Antarctic tunicates of the genera *Aplidium* and *Synoicum* were collected in the Eastern Weddell Sea between 280 m and 340 m depth during the ANT XXI/2 cruise of R/V Polarstern (AWI, Bremerhaven, Germany), from November 2003 to January 2004, by using Bottom and Agassiz Trawls. Individual colonies of each species from a single collection site and trawl were grouped together as a single sample for further experimentation and analysis (Table 1). A portion of each sample was conserved and pictures of living animals were taken on board for further taxonomical identification at the University of Alicante (Spain). The remaining material was frozen at −20 °C, and transported to the laboratory at the University of Barcelona until processed.

**Table 1:** Ascidian samples collected during the Antarctic cruise on board the R/V Polarstern (ANT XXI/2) in 2003 in the Eastern Weddell Sea (Antarctica). B&W: Black & White; O: Orange; Br: Brown morphs; AGT: Agassiz Trawl; BT: Bottom Trawl.

Ascidian species name and code number	Latitude	Longitude	Gear	Depth (m)
*Aplidium falklandicum* Millar, 1960 (1)	70°57.00′ S	10°33.02′ W	BT	332.8
*Aplidium falklandicum* Millar, 1960 (2)	70°55.92′ S	10°32.37′ W	AGT	288
*Aplidium falklandicum* Millar, 1960 (3)	70°56.67′ S	10°32.05′ W	BT	302.4
*Aplidium falklandicum* Millar, 1960 (4)	70°57.11′ S	10°33.52′ W	BT	337.2
*Aplidium fuegiense* Cunningham, 1871	71°7′ S	11°26′ W	AGT	228.4
*Aplidium meridianum* (Sluiter, 1906) (1)	70°56.42′ S	10°31.61′ W	BT	284.4
*Aplidium meridianum* (Sluiter, 1906) (2)	71°04.30′ S	01°33.92′ W	BT	308.8
*Aplidium millari* Monniot & Monniot, 1994	71°04.30′ S	01°33.92′ W	BT	308.8
*Synoicum adareanum* (B&W) (Herdman, 1902) (1)	70°56′ S	10°32′ W	BT	337.2
*Synoicum adareanum* (B&W) (Herdman, 1902) (2)	70°55.92′ S	10°32.37′ W	AGT	288.0
*Synoicum adareanum* (B&W) (Herdman, 1902) (3)	70°56.42′ S	10°31.61′ W	BT	284.4
*Synoicum adareanum* (Br) (Herdman, 1902)	71°06.44′ S	11°27.76′ W	AGT	277.2
*Synoicum adareanum* (O) (Herdman, 1902) (1 and 3)	70°55.92′ S	10°32.37′ W	AGT	288.0
*Synoicum adareanum* (O) (Herdman, 1902) (2)	70°56′ S	10°32′ W	BT	337.2

### 2.2. Organic Extractions

When possible, colonial tunicates were dissected into external/internal (tunic/visceral), and in one case apical, regions, in order to allocate chemical defenses or particular compounds. Each ascidian sample was exhaustively extracted with acetone at room temperature. After removal of the solvent *in vacuo*, the obtained extract was partitioned into diethyl ether (three times) and butanol (once) fractions. The organic phases of each extraction were dried and weighed, providing the yield of extract per dry mass. The natural tissue concentrations were calculated with respect to the total dry weight (DWT = DW dry weight of the extracted sample + EE ethereal fraction weight + BE butanolic fraction weight). Ether extracts were further used for bioassays and chemical analysis, and butanolic fractions and water residues were kept for future studies (Table 2).

**Table 2:** Data of lipophilic Et_2_O extracts and isolated metabolites from the studied Antarctic ascidian samples. [N_EE_]: Natural tissue concentration in mg of dry diethyl ether extract (EE) weight per g of the total dry weight (DW) of the sample; API: Apical part; EXT: External part; INT: Internal part. B&W: Black & White, O: Orange, Br: Brown morphs.

Species name, sample code and body part	[N_EE_] (mg g^−1^ DW)	Isolated metabolites
*Aplidium falklandicum* 1	42.00	Meridianins (A–G) ^a^ + (I–U) ^b^
*Aplidium falklandicum* 2 EXT	57.23	Meridianins (A–G) ^a^
*Aplidium falklandicum* 2 INT	79.3	Meridianins (A–G) ^a^
*Aplidium falklandicum* 3 EXT	47.60	Meridianins (A–G) ^a^
*Aplidium falklandicum* 3 INT	128.40	Meridianins (A–G) ^a^
*Aplidium falklandicum* 4 EXT	23.80	Meridianins (A–G) ^a^
*Aplidium falklandicum* 4 INT	19.40	Meridianins (A–G) ^a^
*Aplidium fuegiense* EXT	15.12	Rossinone B
*Aplidium fuegiense* INT	85.10	Rossinone B + (derivatives) ^c^
*Aplidium meridianum* 1	128.51	Meridianins (A–G) ^a^
*Aplidium meridianum* 2	79.36	Meridianins (A–G) ^a^
*Aplidium millari* EXT	39.31	-
*Aplidium millari* INT	81.60	-
*Synoicum adareanum* (B&W) 1 EXT	20.04	-
*Synoicum adareanum* (B&W) 1 INT	33.09	-
*Synoicum adareanum* (B&W) 2 API	55.69	-
*Synoicum adareanum* (B&W) 2 EXT	18.12	-
*Synoicum adareanum* (B&W) 2 INT	27.31	-
*Synoicum adareanum* (B&W) 3	20.88	-
*Synoicum adareanum* (Br)	36.83	-
*Synoicum adareanum* (O) 1	20.41	-
*Synoicum adareanum* (O) 2 EXT	28.02	-
*Synoicum adareanum* (O) 2 INT	26.43	-
*Synoicum adareanum* (O) 3 EXT	30.71	-
*Synoicum adareanum* (O) 3 INT	66.04	-

^a^ Meridianin mixtures A–G from our samples were not analyzed separately in the current study and are only indicative of the presence of the mixture; ^b^ Meridianins I–U could be present in trace amounts in other meridianin-containing samples, which were not analyzed in more detail due to the lack of enough biological material; ^c^ Rossinone B derivatives 2,3-epoxy-rossinone B, 3-epi-rossinone B, 5,6-epoxy-rossinone B.

### 2.3. Purifications and Chemical Analysis

Diethyl ether (Et_2_O) extracts were screened by Thin Layer Chromatography (TLC), using Merck Kieselgel plates (20 × 10 cm and 0.25 mm thick), and light petroleum ether/diethyl ether (1:0, 8:2, 1:1, 2:8, 0:1) and chloroform/methanol (8:2) as eluents. The plates were developed with CeSO_4_. Four conspicuous UV-visible bands at *R*_f_’s; 0.65, 0.57, 0.45 and 0.21 (light petroleum ether/diethyl ether 2/8) with CeSO_4_ reaction were observed in the *Aplidium fuegiense* INT sample, coinciding with the four meroterpenoid containing fractions. Moreover all fractions pertaining to samples from the species *A. falklandicum* and *A. meridianum* from internal and external regions revealed a yellowish blatant UV-visible band at *R*_f_’s; 0.63 (chloroform/methanol 8/2) with CeSO_4_ reaction, which corresponded with the fraction composed of the alkaloid mixture of meridianins A–G. Extracts were further fractionated on both Sephadex LH-20 and silica gel (Merck Kieselgel 60, 0.063–0.200) columns by using chloroform/methanol 1:1 and a gradient of petroleum ether/diethyl ether as eluent respectively. ^1^H-NMR spectroscopic analyses were carried out to determine pure products or mixtures. Fractions composed of a mixture of molecules were further purified with TLC using preparative (SiO_2_) plates Merck Kiesegel 60 F_254_ (0.50 and 1.00 mm) and HPLC (Shimadzu with LC-10ADVP pump and SPD-10AVP UV detector) using reverse-phase semipreparative columns (Supelco Discovery^®^ C_18_, 25 cm × 46 mm, 5 µm, and 25 cm × 10 mm, Phenomenex, Kromasil C_18_) and water/acetonitrile and methanol/water 70:30 as solvent (flux 2 mL/min). Subfractions from *A. falklandicum* 1 were additionally passed through an Orbitrap LC-MS/MS showing the presence of minor derivative meridianin metabolites.

### 2.4. Spectral Analysis of the Natural Products

The isolated pure compounds were subjected to spectral analysis with NMR, UV, as well as MS spectrometry. Optical rotation measurements were performed on a Jasco DIP-370 polarimeter, using a 10 cm long cell. The ^1^H- and ^13^C-NMR spectra were recorded on Bruker Avance DRX-400, Bruker DRX-600 equipped with in inverse TCI CryoProbe, and Bruker DRX-300 spectrometers. Chemical shifts were reported in ppm and referred to CDCl_3_ and CD_3_OD as internal standard (δ 7.26 and 77.0 ppm for CDCl_3_ and δ 3.34 and 49.0 ppm for CD_3_OD). The ESIMS and EIMS spectra were obtained on a Micromass Q-TOF Micro™ spectrometer connected to a Waters Alliance 2695 HPLC chromatograph, on a Thermo LTQ-Orbitrap Discovery connected on a Accela Thermo Fischer HPLC system, and on a HP-GC 5890 series II spectrometer, respectively. The IR and UV spectra were recorded on a Bio-Rad FTS 155 FTIR and an Agilent 8453 spectrophotometer respectively. The spectral data of compounds isolated were compared with the data reported in the literature [25,28,29]. More detailed data on the chemical procedures may be found elsewhere [13,40].

### 2.5. Feeding Deterrence Assays with Sea Stars

Alive individuals of the voracious, eurybathic, Antarctic sea star *Odontaster validus*, with omnivorous habits and a circumpolar distribution [46] were captured for bioassays at Port Foster Bay in Deception Island, South Shetland Islands (62°59.369′ S, 60°33.424′ W). Captures took place during three campaigns: ECOQUIM-2 (January 2006), ACTIQUIM-1 (December 2008–January 2009) and ACTIQUIM-2 (January 2010). Collection was done by scuba diving from 3 to 17 m depth (*n* > 1300), with the sea stars sizing between 4.5 and 10.5 cm diameter. This asteroid is a model macropredator in many Antarctic feeding deterrence studies (for review see [52]). The sea stars were maintained alive in large tanks with fresh seawater at the Spanish Base BAE “Gabriel de Castilla” (Deception Island), and starved for five days. The bioassays included 10 replicates each, hence, 10 containers filled with 2.5 L of seawater, accommodating one sea star individual. Each asteroid was offered one shrimp food item (5 × 5 × 5 mm and 13.09 ± 3.43 mg of dry mass) that could be fully gobbled, and treatment and control experiments were run simultaneously. This methodology is described in previous papers [53,54]. Control shrimp feeding cubes (12.4% protein, 9.1% carbohydrates and 1.5% lipids, and 17.8 KJ g^−1^ dry wt and 4.1 KJ g^−1^ wet wt, by Atwater factor system [55]) were treated with solvent alone, whereas treatment ones contained natural concentrations of lipophilic Et_2_O extracts or isolated compounds from Antarctic ascidians (Table 2). The extracts or isolated compounds were previously diluted in diethyl ether, and the solvent was evaporated under a flow hood. Previous feeding acceptability studies with asdicians have used several parameters to normalize natural concentrations: volume [56], wet or dry biomass, for biting and no-biting predators [12,13]. In our study, considering sea star extraoral feeding, extruding the cardiac stomach and bolting down whole shrimp pieces [48], dry weight seemed a good approximation for assessing “defense per shrimp feeding cube”. Moreover we chose dry weight because the water content may have produced remarkable deviations in volume and wet weight.

Furthermore, the isolated compound rossinone B and a fraction containing the mixture of meridianins A–G were also assayed at their corresponding sample natural concentrations, which were 4.8 and 19.11 mg g^−1^ dry weight respectively. After 24 h, the number of shrimp items eaten for each test was recorded, and the remaining (not eaten) were frozen for extraction and checked by TLC to ensure the presence of the extracts or compounds, which was always the case. Products contained in diethyl ether extracts are not hydrophilic, hence diffusion to the water column is theoretically implausible, especially in the cold (<1 °C) Antarctic water. Feeding repellence was statistically evaluated with Fisher’s Exact tests for each treatment assay referred to the simultaneous control [57]. After experimentation the stars were returned to the sea.

### 2.6. Feeding Preference Assays with Amphipods

The abundant, eurybathic Antarctic lysianassoid amphipod *Cheirimedon femoratus*, with devouring omnivore-scavenger feeding habits and circumpolar distribution [47], was used for our experiments according to our recently described protocol [58]. Hundreds of individuals were captured between 2 and 7 m depth by scuba diving with fishing nets, and also by displaying baited traps with canned sardines along the coastline of the Antarctic Spanish Base (BAE) during the campaign ACTIQUIM-2 in January 2010. Artificial caviar-textured foods (pearls) were prepared with 10 mg/mL alginate aqueous solution along with 66.7 mg/mL of concentrated feeding stimulant (Phytoplan; 19 KJ g^−1^ dry wt). The powdered food was mixed into the cold alginate solution with a drop of green or red food coloring (see below), and introduced into a syringe without a needle. The mixture was then added drop-wise into a solution of 0.09 M (1%) CaCl_2_ solution where it polymerized forming pellets 2.5 mm Ø (3.3% protein, 1.36% carbohydrates and 1.3% lipids, and 18 KJ g^−1^ dry wt and 1.5 KJ g^−1^ wet wt by Atwater factor system [55]). For extract-treated pearls, ascidian Et_2_O extracts were added at a quantity corresponding to their natural concentration with respect to the dry weight of the food pearls. They were dissolved in a minimum volume of diethyl ether to totally wet the dehydrated food, and the solvent was left to evaporate (Table 2). Control pearls were prepared similarly with solvent alone. Rossinone B and the meridianin mixture were tested too at their sample natural concentrations (see above).

Alive organisms were maintained in 8 L aquaria and were starved for 1–2 days. Every assay consisted of 15 replicate containers filled with 500-mL of sea water and 15 amphipods each, which were offered a simultaneous choice of 10 treatment and 10 control pellets of different colorations (20 food pearls in total), green or red easily distinguishable. The colors for treatment or control pearls were randomly swapped throughout the experimentation period, and previous trials confirmed the null effect of the different colorations in feeding preferences (*P* = 0.4688, n.s.). The assays finished when approximately one-half or more of either food types had been consumed (five pearls of either control or treatment food types), or 4 h after food presentation. The number of consumed and not consumed pearls of each color (control or treatment) was recorded for each replicate container. Since our feeding trials were short in time and performed in very cold water (≈1 °C), autogenic alterations were unlikely. In fact, prior trials had shown that no autogenic changes occurred. Moreover, since food consumption was calculated according to the number of items and not to subtle differences in weight or volume before and after testing, there was no need to run “controls” in the absence of feeders for changes unrelated to consumption [58,59]. Statistics were calculated to determine feeding preference of treated pearls with respect to the paired controls to consequently establish unpalatable activities. Exact Wilcoxon tests were applied using R-command software. Uneaten treatment pearls were preserved for extraction and TLC analysis, to check for possible alterations in the extracts. No major changes were observed. Once testing was over the amphipods were returned to the sea.

### 2.7. Antibacterial Tests against a Sympatric Marine Antarctic Bacterium

These assays were intended to assess antibiotic properties within the ascidian extract, as well as that of the purified compounds rossinone B and the meridianin mixture (A–G) towards an unidentified sympatric marine bacterium. The bacterium was isolated from a seawater sample collected at 3 m depth at Crater 70 area, in Port Foster Bay, Deception Island (Antarctica). A 1 mL alliquot of the seawater sample was transferred into Difco™ marine broth 2216 (Difco Laboratories), grown for 24 h at 18–20 °C, and subsequently cultured in Difco™ marine agar 2216 (Difco Laboratories). The obtained individual bacterial colonies were then isolated, and the strain exhibiting the best growth was chosen for our experiments. A seawater subsample in 7% glycerol filtered-sterilized seawater, as well as a culture of the selected bacterium strain were frozen at −20 °C and shipped to the University of Barcelona for further identification, which unfortunately was unsuccessful. Rinse broth was then inoculated with pure cultures of the selected strain and incubated at 18–20 °C until optimal growth (slight turbidity corresponding to No. 0.5 McFarland scale; equivalent to 10^−8^ cfu/mL). A 0.1 mL suspension of bacterial culture was spread evenly onto marine agar plates. Each Petri dish was divided into six regions: three regions for testing the extracts or isolated compounds in triplicate; another one for the positive control with antibiotic activity; plus two regions for the negative controls, one with and one without solvent. The positive control was chloramphenicol, while negative controls consisted of 20 µL solvent alone, in this case, diethyl ether for the extracts and the rossinone B and methanol for the meridianin fraction. Paper antimicrobial assay disks (BBL Microbiology Systems) Ø 6 mm soaked with the corresponding testing extracts or pure products (rossinone B, meridianin mixture) previously dissolved in 20 µL solvent carrier, or control disks, were placed in the middle of each testing region in the inoculated Petri dishes. Extract and compound amounts added to the disks correspond to natural concentrations calculated as reported below (Table 2). After incubation for one day at 18–20 °C, inhibition halos were measured to determine antibiotic activities. When the diameter of the inhibition zones was larger than 7 mm Ø, it was considered active [60].

## 3. Results

### 3.1. Ascidian Samples and Organic Extractions

Colonies, zooid individuals and larval morphology allowed the identification of our samples as *A. falklandicum*, *A. fuegiense*, *A. meridianum*, *A. millari* and *Synoicum adareanum*. This last species presented three different morphs referred to as: black and white (B&W), brown (Br) and orange (O), clearly distinguishable (Table 1). In total 15 tunicate samples, each consisting of several colonies, yielded 25 diethyl ether extracts that were used for ecological and chemical analysis (Table 2).

### 3.2. Chemical Analysis of the Natural Products

**Figure 1:**
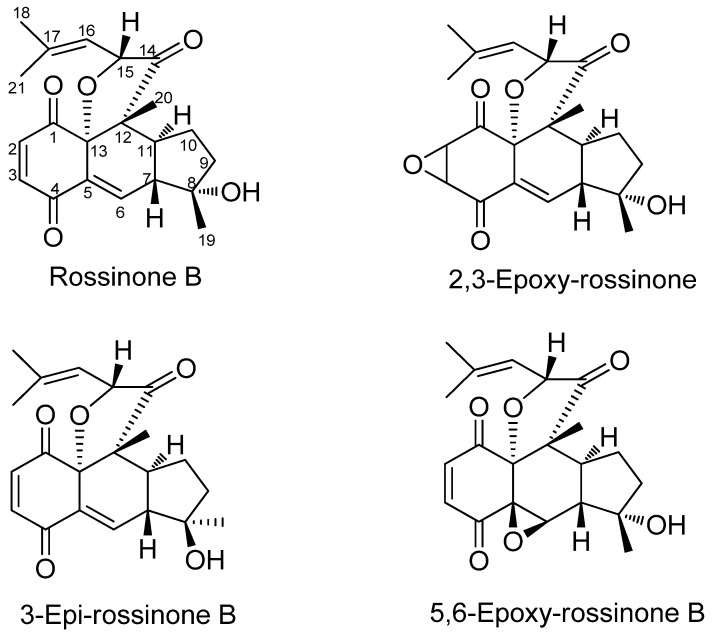
Chemical structures of the rossinone compounds purified from *Aplidium fuegiense*: rossinone B and the three derivatives 2,3-epoxy-rossinone B, 3-epi-rossinone B, 5,6-epoxy-rossinone B.

**Figure 2:**
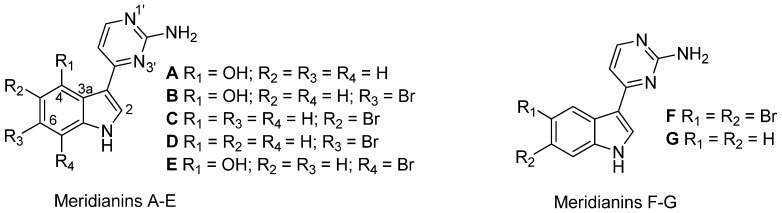
Chemical structures of the meridianin compounds (A–G) purified from *Aplidium falklandicum* and *A. meridianum*.

Four meroterpene derivatives, of the class of the cyclic prenyl quinones, rossinones B and the three derivatives 2,3-epoxy-rossinone B, 3-epi-rossinone B, 5,6-epoxy-rossinone B (Figure 1), were isolated from the Et_2_O lipophilic internal fraction of the colonial Antarctic tunicate *Aplidium fuegiense* (*A. fuegiense* INT). In contrast, the tunic of the sample (*Aplidium fuegiense* EXT) possessed very small quantities of rossinone B, but lacked the other minor rossinone meroterpene-related products. Rossinone B, which was first reported in an *Aplidium* sp. ascidian from the Ross Sea, Antarctica [29], was the major metabolite of this family of compounds. Rossinones B and related derivatives (Figure 1) were also recently described as part of our chemical investigations [40]. Furthermore, all the extracts from internal viscera and external regions from samples of the species *A. falklandicum* and *A. meridianum* revealed the presence of the known meridianins A–G (Figure 2). The purified meridianin fraction from the sample *A. falklandicum* 1 was used in the sea star assay. Finally, a group of twelve unknown minor meridianin derivatives (I, J, J′, L, O, P, Q, R, R′, S, T and U) with combinations of bromide, chloride, and hydroxy groups, as well as two unknown dimeric derivates from the majoritary meridianins A and B (or E) were detected by means of an Orbitrap LC-HRMS-MS [61] from the sample *A. falklandicum* 1 (see Supplementary Material).

### 3.3. Feeding Deterrence Assays with Sea Stars

**Figure 3:**
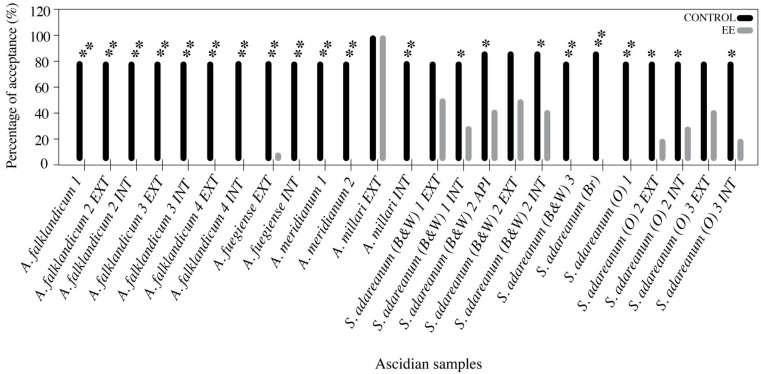
Bar diagram displaying the results in the feeding repellence bioassays with the sea star *Odontaster validus* performed with lipophilic Et_2_O extracts from Antarctic colonial ascidians, showing the paired results of control and extract treated shrimp cubes for each test and representing the percentage of acceptance. * significant differences (*P* < 0.05), ** significant differences (*P* < 0.01), with control as preferred food (Fisher’s exact test).

All five ascidian species and 15 samples demonstrated the presence of chemical defenses. Twenty-one of the lipophilic Et_2_O fractions tested caused significant (*P* = 0.01 or *P* = 0.05) feeding repellence against the sea star *O. validus* at natural concentrations according to the Fisher’s Exact test. Control assays using shrimp feeding cubes with solvent alone displayed a minimum acceptance of eight cubes out of ten. In 14 experiments the lipophilic fractions caused an absolute rejection by the sea stars (*P* = 0.01). On the other hand, only four samples from the external tunics of *Aplidium millari*, *Synoicum adareanum* (B&W) 1 and 2 and *S. adareanum* (O) 3 were accepted (Figure 3). Regarding the tests conducted with isolated metabolites, both the rossinone B (*P* < 0.001), as well as the mixture of meridianins A–G (*P* < 0.001) showed potent deterrence against the asteroid at their natural concentrations. In both cases the consumption was 0 out of 10 compound-treated cubes, whereas the simultaneous control tests had a ratio of eight items eaten out of ten.

### 3.4. Feeding Preference Assays with Amphipods

**Figure 4:**
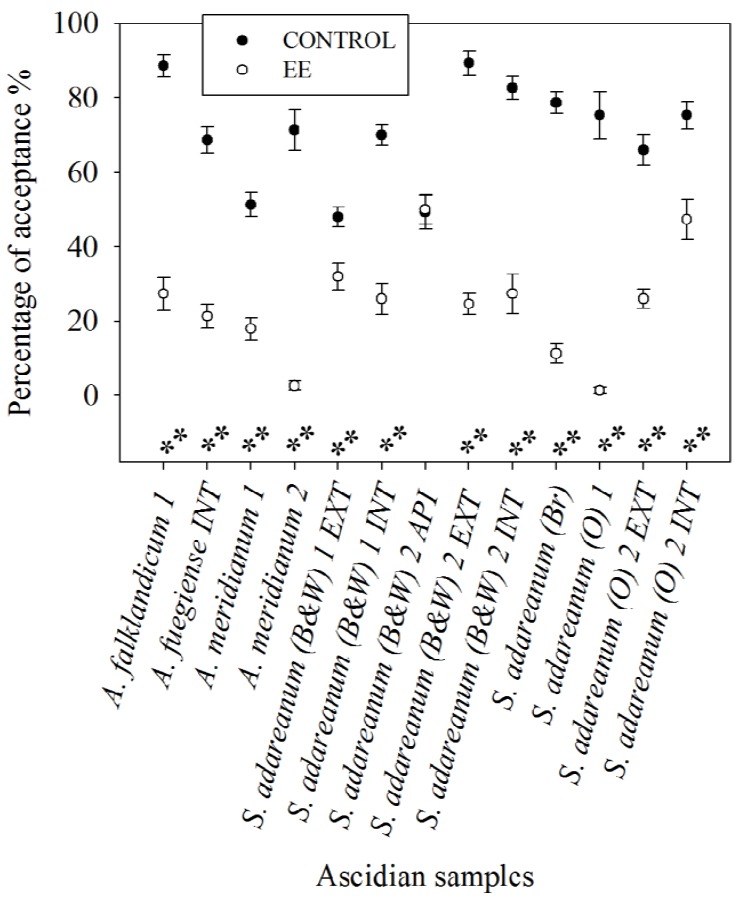
Scatter plot diagram showing the results in the feeding preference bioassays with the amphipod *Cheirimedon femoratus* conducted with lipophilic Et_2_O fractions from Antarctic colonial ascidians. The paired results of control and extract treated food pearls are displayed for each test as the mean percentage of acceptance and standard error bars. ** significant differences (*P* < 0.01) with control as preferred food (Exact Wilcoxon test).

In the preference experiments towards the amphipod *Cheirimedon femoratus* the four species tested, represented by nine samples were shown to contain repellent compounds. In fact, all the fractions assayed except one (12 out of 13) revealed remarkable feeding unpalatable activity (*P* < 0.01) at natural concentrations according to the Wilcoxon Exact test. The amphipod devoured control food pearls at impressive high rates, and regardless of its gregarious behavior unpalatabilities were evident. Actually most of the extracts that yielded deterrence in this assay were strongly rejected and not ingested at all when they were presented included in alginate pearls. Only the apical ethereal fraction (API) from the ascidian *Synoicum adareanum* (B&W) 2, was palatable contrasting with basal-external and visceral extracts (EXT and INT), which were remarkably repellent (Figure 4). In addition, the amphipod significantly rejected food pearls treated with rossinone B (*P* < 0.01) or meridianin (A–G) mixture (*P* < 0.001), with respect to the controls. 

### 3.5. Antibacterial Tests against a Sympatric Marine Antarctic Bacterium

The isolated mixture of meridianins from *Aplidium flaklandicum* 1 caused strong growth inhibition (active (+++) in the three replicates >10 mm Ø inhibition halo) on cultures of an unidentified sympatric Antarctic marine bacterium, as did the positive controls with chloramphenicol. On the contrary none of the extracts assessed from our ascidian samples, nor the rossinone B inhibited the bacterium in our laboratory assays, similarly to what was observed in the solvent negative controls.

## 4. Discussion

### 4.1. Incidence and Allocation of Chemical Defenses against Predation

Antarctic ascidians thrive in environments where predation pressure, mostly driven by invertebrate consumers, is intense [1,45]. Still, only seldom has it been demonstrated that natural products are responsible for chemical defense in ascidians [4,56,62]. Moreover, these animals exploit inorganic acids against sea star predators (especially colonial ascidians) and the protection afforded by a tough tunic (especially solitary ascidians) [12,56,63]. Our findings complete this map by showing that organic chemical defense is largely used in these ascidians, since all our samples possess repellent metabolites (Figure 3 and Figure 4). The specimens and, in general, the species analyzed in this study were free of evident epibionts and lacked mechanical protection [51]. Likewise, bioaccumulation of acids or heavy metals has not been reported within their tunic, nor in closely related species of the family Polyclinidae [9,10,64], which in fact report absence of bladder cells [65]. These facts put forward the need for protection based on organic chemistry. On the other hand, lipophilic partitions have proved to be more actively deterrent than hydrophilic ones in marine organisms [23,52,66], and thus we focused our study on the ether fractions of our specimens. In the past however, only rarely have the chemicals responsible for the unpalatablity been identified. Yet some examples of deterrent metabolites in ascidians include the tambjamines C and F, didemnin B and nordidemnin B, patellamide C, ascididemin, and meridianins A–G [8,13,15,22,23].

*Aplidium falklandicum* and *A. meridianum* possess protective chemicals, the meridianins, which besides deterring the asteroid *Odontaster validus*, have now shown feeding repellence towards the amphipod *Cheirimedon femoratus*. Meridianins are present both in inner and outer tissues, even if they seem to be more concentrated in outer zones [13]. Apart from these two species, a seeming lack of within-specimen defense allocation was detected in *S. adareanum* (O) 2, as has also been observed in other ascidians [38]. Rossinone B was proven to take part in the whole-colony chemical defense of *A. fuegiense*, repelling both sea stars and amphipods, but it was predominant in internal regions.

According to the ODT [41], tunics with low palatability (determined by a combination of energy content, digestibility, chemicals and, pH) are expected when protecting adult stages surpasses the benefits of defending larval ones [4]. In fact, in some colonial species bioactive alkaloid pigments are stored in tunic bladder and pigmentary cells, presumably acting as sunscreens or deterrents [31,38,39]. However, the presence of chemical defenses within the tissues of some Antarctic sponges and ascidians suggests that predators other than sea stars are also relevant, or that the assumptions of the ODT are inappropriate in such a case [13,67,68]. Also, big complex eggs and larvae produced by most compound ascidians are often protected with noxious cyclic peptides and alkaloids, compensating the great investment assigned to reproduction [4,14,15,30]. This outcome explains the presence of deterrents in inner tissues (gonads) in order to produce chemically defended larval stages [11,15]. The predominant internal allocation of defenses in some of our samples is thus not fortuitous. Some tunics have low caloric value with respect to inner tissues, making them already less attractive to predators McClintock [4,11,69]. Besides, colonial ascidians are often able to recover from wounds and rapidly regenerate the damaged tunic [70]. This capacity allows them to address less energy in defending non reproductive regions. On the contrary, solitary species may require better-protected tunics [69]. Pisut and Pawlik [11] found deterrents allocated in the gonads of solitarian species, yet whole-specimen extracts were palatable. This indicated the possession of thick tunics that diluted any deterrence found in viscera and gonads. Our compound asdidians, instead, had thin tunics accounting for a small fraction in the colony, and even if some samples had poorly (or not) defended tunics, whole-colony extracts were always deterrent. Tunics from *A. millari*, *S. adareanum* (B&W) and *S. adareanum* (O) seemed to be less (or not) chemically protected against sea star predation. However amphipod assays, probably due to a greater susceptibility of *C. femoratus* [71], do reflect the existence of deterrents in the tunics, presumably in lesser amounts. The supposed low energetic value of the tunics, along with a weak chemical defense with respect to inner regions may contribute to the overall protection of these colonies against heavy predation, complementing the remaining defensive mechanisms. The lower extract yields produced by most tunics with respect to inner tissue likely reflect these facts (Table 2). Furthermore, this pattern of allocating deterrents into the internal regions was also observed in the distribution of the defensive secondary metabolite rossinone B within the colonies of *A. fuegiense*.

### 4.2. Antibiotic Activity towards Marine Bacteria

Benthic organisms must combat pathogens as well as epibiosis by macro- and microorganisms. More commonly colonial rather than solitary ascidians, have been found to possess agents to prevent this [19,24,72,73,74,75]. Our Antarctic samples however, did not display significant inhibition against a sympatric bacterium strain. This agrees with other surveys of both Antarctic sponges and ascidians, which indicate a general lack of antibacterial chemistry. In Antarctic systems, diatom invasions apparently surpass that of bacteria, suggesting that there might be more selective pressure for chemical defenses against diatom fouling [63,76,77,78]. It was also proposed that bacterial pathogens could be controlled through immune processes in asdicians [63,66]. Rossinone B, which was antimicrobial and antimycotic towards cosmopolitan strains [29], revealed no activity in our assays. Meridianins A, B, C, E, F and G, instead, caused no growth inhibition on allopatric microbes in the past [13]. However, in the present study the meridianin mixture revealed potent activity against an Antarctic marine bacterium suggesting a defensive role against pathogenic or fouling bacteria. Even if whole ascidian extracts seem inoquous, they are composed of a complex mixture of substances (primary and secondary metabolites, and nutrients) that may interfere with some bioactivities. However, if meridianins were to be allocated in compartments, which has not been proved so far, they could then appear in higher concentrations and fulfil this function too. Despite the fact that some biologically active marine natural products serve specific ecological roles [75], others, such as the meridianins, seem to be multipurpose defenses. In the current study, the antibacterial testing, being on a single, unidentified, bacterium isolated from shallow seawater samples, possesses limitations regarding information of ecologically significant antifouling properties. Indeed further investigations should be addressed with deep-water representative bacterial populations.

### 4.3. Variability and Origin of Bioactive Natural Products

Secondary metabolites are more typical of colonial than of solitary tunicates, and have been found in deep as well as in shallow specimens, even if these last ones have been more frequently analyzed. Chemical analyses have been reported for six species of Antarctic ascidians coming from both shallow and deep collections, all of them colonial: *Synoicum* sp., *S. adareanum*, *Aplidium* sp., *A. falklandicum*, *A. meridianum* and *A. fuegiense* [13,40,52,79]. Diyabalanage and co-workers purified a cytotoxic macrolide, palmerolide A, from *S. adareanum* [26]. A dense microbial community was detected on the tunicate and a possible bacterial origin of this polyketide was proposed [80]. Several ecdysteroids (arthropod molting hormones) were also reported from *S. adareanum* [27]. Their presence suggested a potential to defend from arthropod predators through a strategy similar to that found in terrestrial plants, which elaborate ecdysteroids that short-wire molting in phytophagous insects. In our investigation we did not find these metabolites, however this species did exhibit amphipod feeding-avoidance. We must point out that intraspecific polymorphism in colonial ascidians is recurrent [51], and we found three morphotypes for *S. adareanum* among our samples. *S. adareanum* also occurs in two different morphs near Palmer Station, each revealing diverse bioactivities. Moreover, crude extracts of a *S. adareanum* from shallow areas of Anvers Island (western Antarctic Peninsula) were found to lack deterrence towards several sympatric consumers [56], as opposed to our results from deep-water samples. The variable morphologies, bioactivities, and presence of some characteristic metabolites suggest a need for further taxonomical resolution in this species [63].

Ascidians of the genus *Aplidium* are renowned for the variability of the metabolites that they possess: non-nitrogenous compounds are dominated by prenyl quinones, linear or cyclic, and among the nitrogen containing group, nucleosides, cyclic peptides and a high variety of alkaloids can be mentioned [81]. While the majority of ascidian metabolites are amino acid derived [82], the genus *Aplidium* is noted for its propensity to biosynthesize terpene derivatives [81]. The finding of rossinones B and related 2,3-epoxy-rossinone B, 3-epi-rossinone B, 5,6-epoxy-rossinone B in *A. fuegiense*, reflects this outcome, since meroterpenes are typically found in sponges and seaweeds [83]. Rossinones A and B were first isolated from an Antarctic unidentified *Aplidium* from the Ross Sea. While modest bioactivities characterized rossinone A, rossinone B exhibited antileukemic, antiviral, and anti-inflammatory properties [29]. Biosynthetically, cyclic prenylated quinones, such as rossinone B and the three derivatives from this study seem to derive from linear hydroquinones, like rossinone A [84]. Interestingly, neither acyclic hydroquinones nor putative quinone-containing precursors of rossinones were detected in *A. fuegiense* [40].

It would be interesting to find out where all these compounds are synthesized. In other colonial species, special tunic cells (bladder cells; lacking in *Aplidium* and *Synoicum* [65], or pigment cells) concentrate defensive chemicals [36,38,39]. Final metabolites seem to end in storage compartments in the outer tunic, while other intermediate products remain in inner producing tissues (zooids) [38]. This could explain the distribution observed for the rossinone compounds in *A. fuegiense*. Here, rossinone B is the major and most active defensive metabolite. It was found predominantly in inner tissues, but also in the tunic in small amounts. The other minor rossinones (2,3-epoxy-rossinone B, 3-epi-rossinone B, 5,6-epoxy-rossinone B) in contrast, are only present in internal areas of the colony, presumably as precursors. Alternatively, these products could derive from symbiotic microbes. Among the known microorganism-derived products, terpenes are uncommon and indole alkaloids predominate [22,85,86,87,88]. In many species, especially colonial, microsymbionts are usually sited in the tunic [33] (and reviewed in [3,34,35]). The presence of the intermediate products exclusively in the inner tissues [40], suggests that rossinone terpenoids probably do not derive from a microbial source, or at least not from a tunical symbiont.

**Figure 5:**
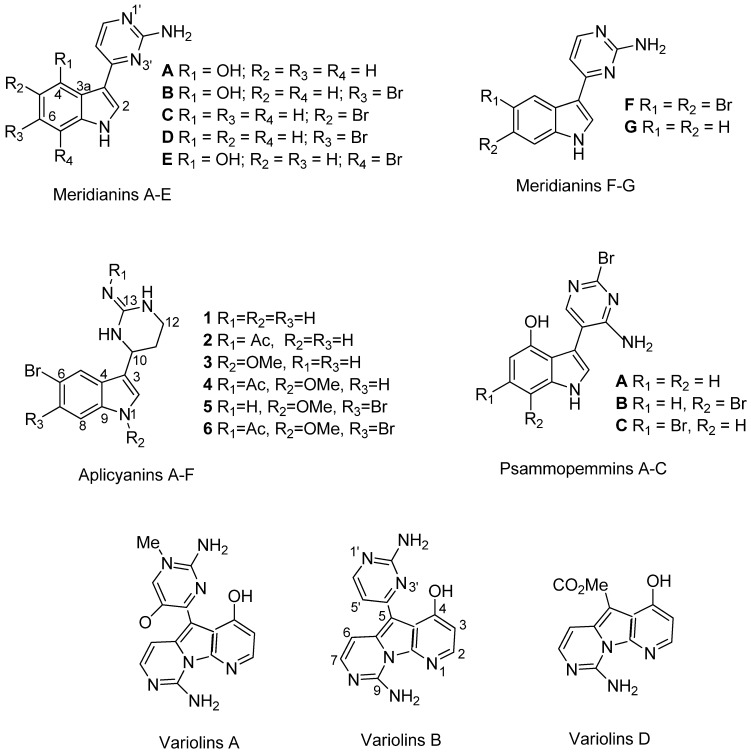
Chemical structures of meridianin-related indole alkaloids obtained from Antarctic marine organisms: Aplicyanins A-F from the ascidian *A. cyaneum*, Psammopemmins A–C from the sponge *Psammopemma* sp. and variolins A, B and D from the sponge *Kirkpatrickia variolosa*.

The meridianins are a family of indole alkaloids with potent cytotoxicity and kinase inhibitory activity, especially meridianins B and E, which are considered an important scaffold for cancer therapeutics [89,90]. Rossinones and meridianins are indeed interesting products for pharmacological research. The new minor meridianins (I–U) (Supplementary Material) and some unreported dimeric derivates indicate that the meridianins constitute a bulky group of alkaloids very high in concentration and in diversity. Many deterrents appear as a family of related metabolites, which are effective as a mixture, but often also as isolated forms, such is the case of both tambjamines and meridianins [13,22]. Colonies of *A. falklandicum* and *A. meridianum* have external yellowish pigmentation, like the fraction containing the mixture of meridianin compounds (A–G). Meridianins could be photoprotective, as proposed for other species containing bright-colored alkaloids [30,39,87]. Nonetheless, in our particular case since the specimens were collected at <200 m depth where light no longer penetrates, concepts such as photoprotection and UV-screening are irrelevant. Thus the meridianins at this point could be considered as relict pigments. Antarctic benthic invertebrates have no apparent reason to have warning bright colorations in a system where grazing pressure by visually oriented predators, such as fish, is generally lacking. Yet many organisms are highly pigmented and the related bioactive pigments are themselves feeding deterrents and/or antifoulants. The role of coloration here may respond to an evolutionary selection for, or retention of, pigmentation driven by predation pressure. As a result, relict pigments originally selected by aposematism or UV-screening are conserved because of their defensive properties, even in species living in deep-waters where coloration has no apparent sense. Among these bioactive pigments are the variolins from *Kirkpatrickia variolosa*, discorhabdins from *Latrunculia apicalis*, suberitenones from *Suberites* sp. and those from *Dendrilla membranosa* (reviewed in [66,87]).

The meridianins have been obtained from geographically distinct Southern Ocean ascidian populations generally collected at more than 100 m depth, and from several species: *A. meridianum*, *A. falklandicum* [13,25,28] and *Synoicum* sp. [91]. The two *Aplidium* species though, are being revised and might actually be synonymised in the future [92]. It is intriguing, however, that meridianin D, although a major metabolite, has been repeatedly isolated from *A. meridianum* samples but not from other species, maybe representing a specific feature [13,25,28,91]. Furthermore meridianin A, B and E, have been recently reported to correspond to the so-called psammopemmins A, C and B respectively, described from the Antarctic sponge *Psammopemma* sp. [91,93]. Thus, the broad existence of these alkaloids in Antarctic animals, along with those of the closely related aplicyanins and variolins [94] could respond, either to symbiotic associations and microbe elaboration, or to co-evolution and retention of biosynthetic pathways of metabolites with adaptive functions (Figure 2 and Figure 5). The versatility of these alkaloids in terms of ecological functionality justifies the broad acquisition of these metabolites by a number of Antarctic species [13,25,28,91,93]. A similar situation occurs with the tambjamine alkaloids, found in bryozoans and ascidians (and molluscs feeding on them) from a variety of habitats, which are moreover related to bacterial pigments [22,86]. Meridianins, as aminopyrimidine indoles might derive from a pyrimidic base by connection of a pyrimidine ring onto an indole system [90]. In fact, 2-deoxythymidine was detected in our *Synoicum* and *Aplidium* ascidian samples. Actually, nucleoside derivatives are frequent in *Aplidium* species [81], and maybe precursors of complex secondary metabolites [21].

## 5. Conclusions

Defensive strategies of some shallow temperate and Antarctic colonial ascidians were previously proposed to be highly variable, and seldom to be based on organic chemistry. In contrast, our results with deep-water specimens indicate that selective pressures for chemical defenses against predation are important in the evolution of Antarctic colonial ascidians, since all the species here analyzed had effective lipophilic deterrents. Moreover many of the samples tended to store repellent agents in the internal regions of the colony, and in particular this was observed in the species *A. fuegiense*, *A. millari*, and *Synoicum adareanum* orange and B&W colorations. In fact, the isolated deterrent metabolites analyzed from *Aplidium* specimens seem to have different patterns of within-colony allocation, which along with the diverse molecule-type may suggest also a distinct origin. Whereas the rossinones were characteristic of internal tissues, where their synthesis is likely to occur, the meridianins displayed greater concentrations in the outer regions presented in one of our previous investigations. The meridianins, moreover, have been found in several ascidian species of the genera *Aplidium* and *Synoicum* and in sponges from Antarctic waters, leading to suspicions that they might represent relict pigments retained for their multifunctional defensive roles. As with many other bioactive alkaloid pigments the meridianins could be hypothesized to derive from symbiotic microbes. In agreement with other Antarctic studies with ascidians and sponges, our crude ether extracts exhibited low prevalence of antibacterial properties, even though the meridianin fraction did show inhibitory activity against a sympatric bacterium. This represents one of the very few studies in which deterrents were identified and localized in Antarctic deep-water ascidians. Further investigations should be undertaken to increase our knowledge of the nature and function of chemical defenses in the Southern Ocean, and to compare shallow and deep-water habitats in relation to defensive adaptations.

## Supplementary Files

Supplementary File 1: PDF-Document (PDF, 183 KB)
