# Supplementary material for: Natural Products from Antarctic Colonial Ascidians of the Genera Aplidium and Synoicum: Variability and Defensive Role
Source: Mar Drugs. 2012 Aug 20;10(8):1741–64. doi: 10.3390/md10081741 (PMC3447337; doi:10.3390/md10081741)
Supplement: Supplementary File 1: — PDF-Document (PDF, 183 KB) [file marinedrugs-10-01741-s001.pdf]

Supplementary Material

Figure S1. Proposed chemical structures of the new minoritary meridianins I–U detected by LC-HRMS/MS from *Aplidium falklandicum* 1.

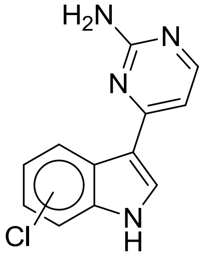

Meridianin I

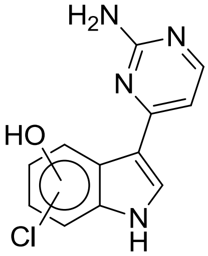

Meridianin J
Meridianin J'

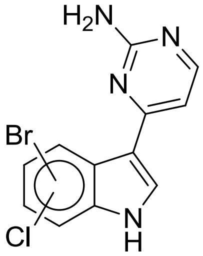

Meridianin K

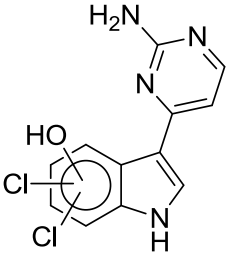

Meridianin L

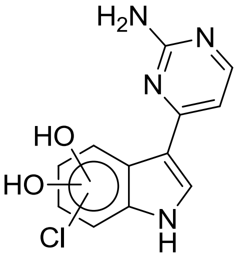

Meridianin O

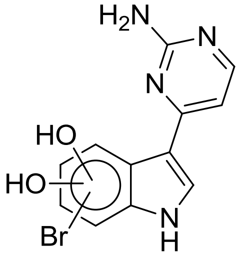

Meridianin P

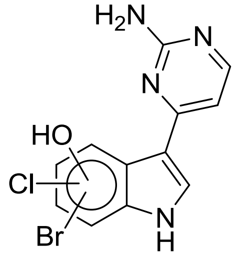

Meridianin Q

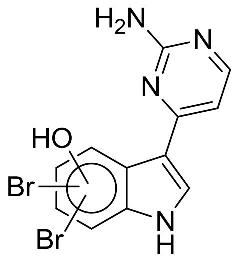

Meridianin R
Meridianin R'

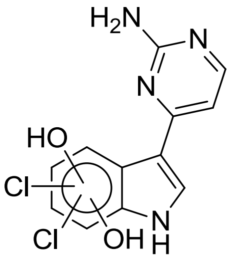

Meridianin S

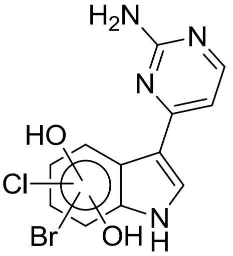

Meridianin T

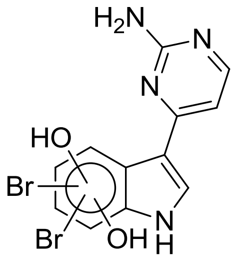

Meridianin U
